# Evaluation of LSTM vs. conceptual models for hourly rainfall runoff simulations with varied training period lengths

**DOI:** 10.1038/s41598-025-96577-4

**Published:** 2025-05-06

**Authors:** Mohamed M. Fathi, Md Abdullah Al Mehedi, Virginia Smith, Anjali M. Fernandes, Michael T. Hren, Dennis O. Terry

**Affiliations:** 1 Department of Civil and Environmental Engineering, Villanova University, Villanova, USA; 2 Department of Civil Engineering, Faculty of Engineering, Fayoum University, Fayoum, Egypt; 3 Department of Earth and Environmental Sciences, Denison University, Granville, USA; 4 Department of Earth Sciences, University of Connecticut, Storrs, USA; 5 Department of Earth and Environmental Science, Temple University, Philadelphia, USA; 6 Department of Civil Engineering, Florida Gulf Coast University, Fort Myers, USA

**Keywords:** Length of calibration dataset, GR4H and GR5H models, One-step ahead prediction, Efficient LSTM models, Hybrid ML and conceptual models, Longer training dataset, Hydrology, Environmental sciences

## Abstract

Accurate high-resolution runoff predictions are essential for effective flood mitigation and water planning. In hydrology, conceptual models are preferred for their simplicity, despite their limited capacity for accurate predictions. Deep-learning applications have recently shown promise for runoff predictions; however, they usually require longer input data sequences, especially for high-temporal resolution simulations, thus leading to increased model complexity. To address these challenges, this study evaluates the robustness of two novel approaches using Long Short-Term Memory (LSTM) models. The first model integrates the outputs of a simple conceptual model with LSTM capabilities, while the second model is a stand-alone model that combines coarse and fine temporal inputs to capture both long and short dependencies. To ensure accuracy and reliability, we utilized a century-long meteorological dataset generated from a sophisticated physics-based model, eliminating any influence of measurement errors. The training phase employed multiple sub-periods ranging from 7- to 50-year, with a separate 50-year subset for validation. Our findings highlight the consistent improvement of both LSTM models with increasing training dataset lengths, while conceptual models show no notable enhancement beyond 15 years of training data. Both LSTM models demonstrate superior performance in capturing the reference flow duration curve, offering a promising pathway for more computationally efficient models for runoff predictions.

## Introduction

High-resolution runoff predictions are essential for understanding the dynamics of hydrologic systems, ultimately leading to effective water resource planning and management. Over the past few decades, rainfall-runoff models have evolved significantly to estimate the streamflow time series across various temporal and spatial scales^1^. These models are generally grouped into three main categories: physics-based, conceptual, and data-driven^2^. The physics-based approach employs the principles of physical processes to simulate the mechanism of hydrologic systems. These physical processes typically include fundamental principles such as conservation of mass, momentum, and energy, as well as detailed simulations of hydrological phenomena like infiltration, percolation, routing, and evapotranspiration^3^. This approach entails longer calibration processes and requires a detailed description of the catchments’ physical characteristics^4^. In contrast, conceptual models provide simplified mathematical representations of the physics principles for hydrological simulations, requiring limited inputs within a computationally efficient framework^5^. Recently, data-driven models have gained significant attention due to their ability to handle complex simulations by capturing the non-linear relationships between the inputs and the outputs without the need for any physical knowledge^6^.

In recent years, numerous Deep Learning (DL) techniques have been utilized in the hydrological field, particularly for predicting runoff responses^5,7^. Among these, Long Short-Term Memory (LSTM), a variant of Recurrent Neural Networks (RNN), has demonstrated high efficacy in handling sequence patterns for time-series modeling^8–10^. LSTM addresses the traditional RNN issues of vanishing and exploding gradients through its integrated structure of input, output, and forget gates^11^. Several studies highlighted the capabilities of LSTM in providing reliable runoff responses^5,6,12^. A comparative analysis was conducted to evaluate the performance of LSTM with other DL techniques, including Artificial Neural Networks (ANN) and Gated Recurrent Unit (GRU) networks, in simulating runoff responses^7^. The results indicated that both LSTM and GRU models performed equally well, significantly outperforming the ANN model. Similarly, the performance of LSTM as assessed in comparison to ANN and M-EIES, a physics-based model, showed that the LSTM model outperformed both models under normal and extreme rainfall conditions^13^. Recently, eight data-driven models were evaluated against the Soil and Water Assessment Tool (SWAT), a complex physics-based model, underscoring the potential of LSTM models in predicting runoff responses^14^.

LSTM models have been used for predicting hydrographs across various time intervals: including 15-min^15^, 1-h^16^, 3-h, and 6-h^7^, daily^14^, and even monthly time step^17^. However, developing high-resolution rainfall-runoff models necessitates the incorporation of long Input Data sequence Length (IDL), which typically requires more complex and computationally expensive models. For instance, various IDLs for monthly runoff predictions were investigated, indicating that a 6-month IDL is sufficient to capture the monthly temporal pattern^18^. Moving to daily rainfall-runoff modeling, a 180-day IDL was used to capture the dynamics of the annual pattern^11^. An IDL of 4320 h was introduced for hourly simulations^19^. The primary challenge of increasing the temporal resolution of the model lies in the necessity to extend the IDL: 6 months, 180 days, and 4320-h values for monthly, daily, and hourly simulations, respectively. A higher IDL typically leads to a more complex model and increased computational expenses. To reduce the required computational time, daily and hourly meteorological data were combined to estimate the hourly runoff values^6^. Instead of employing an 8760-h IDL, they used a combination of a 365-day IDL with hourly IDL trials of 24, 48, and 120 h. While this study demonstrated a noticeable reduction in the IDL compared with the earlier studies; there remains a need to further reduce the IDL duration to enhance the efficiency of DL models. Enhanced model efficiency is critical for improving water resource planning and management practices through reduced training times and accelerated prediction capabilities.

Data-driven models yield remarkable results, but impose substantial demands in terms of data requirements, extensive training periods, time-consuming parameter tuning, and computational resources according to their architecture and complexity^19^. Conversely, conceptual models typically offer superior computational efficiency, requiring significantly less calibration time^20^. Notably, a number of lumped conceptual models have been developed for runoff predictions, demonstrating their potential in hydrological modeling^21^. These models are available at different temporal resolutions, ranging from the annual to sub-hourly time scales^22–25^. The complexity of these models can be defined according to their structure and the number of calibrated parameters. Three representative lumped models were selected to exemplify the conceptual approach, each exhibiting distinct levels of complexity: GR4H, GR5H, FLEX with 4, 5, and 10 calibrated parameters, respectively^21,26,27^. Despite the applicability of both conceptual and data-driven approaches, there is a serious need to investigate the capabilities and limitations inherent in each approach. This assessment will facilitate the formulation of precise recommendations and optimal practices for their implementation in hydrological modeling, thereby enhancing the efficacy of water resource planning and management strategies.

The rainfall-runoff modeling framework has long been constrained by the limited length of available datasets, which hinders the evaluation of model performance. DL techniques generally require large training and testing datasets; however, most of the historical records used for RNN applications span only a few years to a decade. For instance, the testing phase of three DL models was conducted using only 2 years of data^6^. Similarly, a 5-year testing dataset was employed to evaluate the accuracy of a data-driven model^28^. Even for the applications on large-scale datasets such as CAMELS, only 10 years of data were used for the testing step^19^. To address this gap, this study utilizes a longer meteorological dataset generated based on a sophisticated physics-based model^29^. This step aims to investigate the impact of training dataset length on the performance of the DL and conceptual models in predicting the runoff responses from hydrological forcing variables.

Ultimately, this study tests and compares the efficiency and accuracy of novel LSTM models versus conceptual models in predicting high-resolution hourly runoff responses. This is accomplished through: (a) developing LSTM models with more efficient architectures using limited multi-scale inputs, (b) evaluating the accuracy of the developed models by comparing them with a set of efficient conceptual models, and (c) assessing the impact of calibration data length on model performance using a 100-year hourly dataset.

## Study area and data sources

**Fig. 1:**
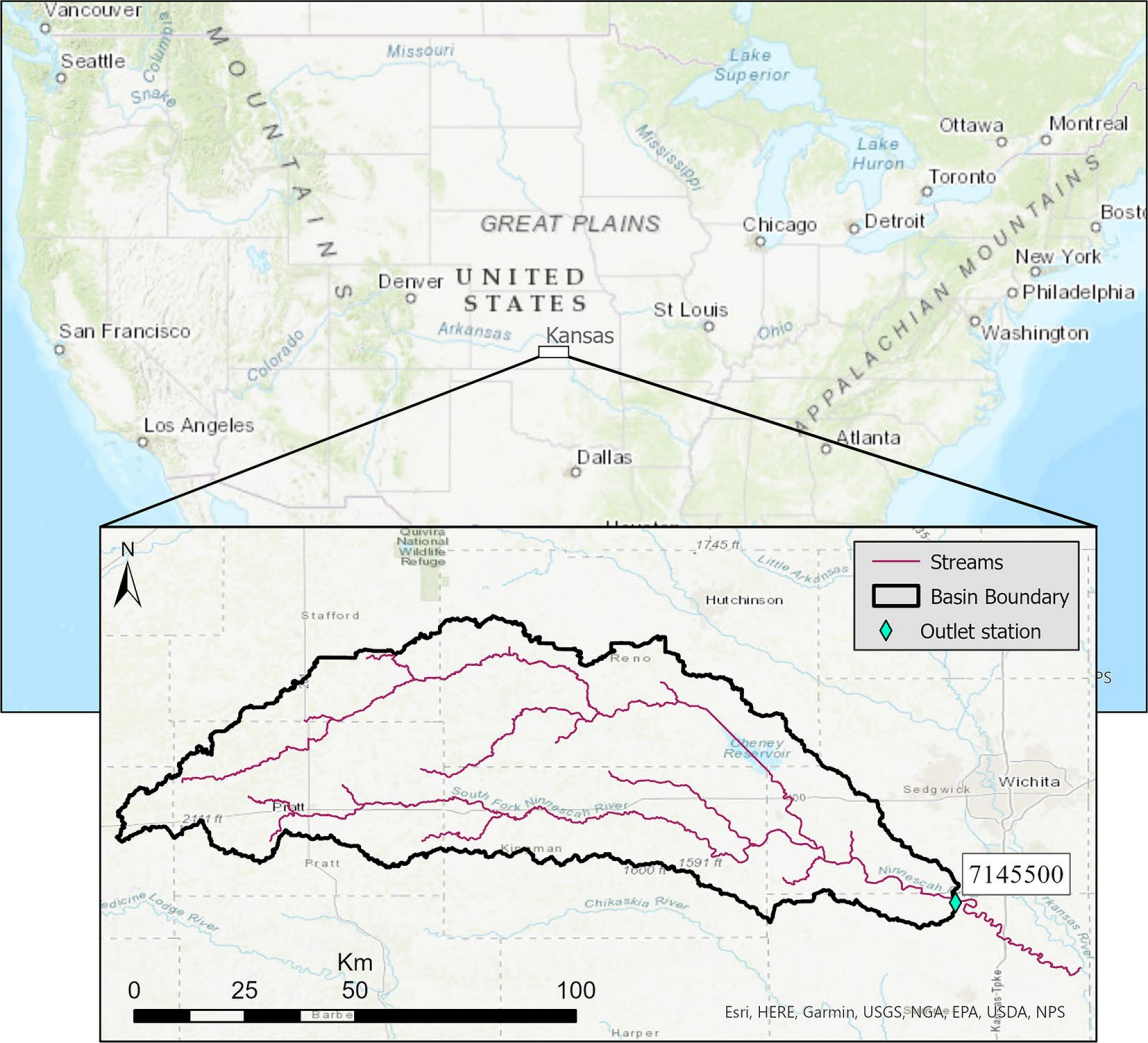
Location of Ninnescah River watershed at USGS station 07145500^32^. The map was generated using ArcGIS Pro 3.3 software.

The Ninnescah River, located in south central Kansas, USA, was investigated as a test case application for this study. As a tributary of the Arkansas River, it originates from two forks, the north and south, before meandering to the east-southeast (Fig. 1). The drainage area of the outlet (at USGS gage 07145500) is approximately 5500 km^2^. The basin experiences an average annual precipitation depth of around 800 mm^30^. The observed hydrographic data exhibits high winter discharge peaks and low baseflow discharges during the summer months^31^.

This study employs a metrological dataset generated through a novel approach, which was developed to construct a long hydrograph series^29^. This methodology conserves the statistical, frequency, and stochastic properties inherent in observed hydrographs for the basin under study. This innovative approach comprises three key steps: (a) generating a daily precipitation series using a stochastic weather generator; (b) disaggregating the generated daily precipitation series into a finer hourly timescale; (c) estimating the runoff responses through a continuous rainfall-runoff transformation simulation using HEC-HMS model, ultimately producing a precise long-term hydrograph. This approach was used to generate a high-resolution long hydrograph that exhibits a satisfactory correlation with the observed series according to statistical criteria and the Flow Duration Curves (FDCs)^29^. Their contribution concluded with generating a century-long dataset comprising precipitation, Potential Evapotranspiration (PET), and runoff responses at hourly resolution. While the extensive temporal coverage is integral to this analysis, it is essential to acknowledge that the dataset is derived from model simulations rather than direct ground-based observations, which may introduce inherent limitations. Nonetheless, this long-term scale is crucial for examining the influence of calibration dataset length on model performance. This approach enables the assessment of various lengths of calibration datasets, leading to a better understanding of best practices when dealing with short or long datasets and identifying which models are most suitable under each scenario.

## Methodology

### Conceptual models

GR4H^33^, GR5H^26^, and FLEX^22^ are commonly used computational models in rainfall-runoff modeling, particularly on an hourly time scale. The GR4H model, an adapted hourly version of the daily model GR4J, employs only two reservoirs and four calibrated parameters to simulate the rainfall-runoff dynamics^27,33^. The GR5H model, an extension of GR4H, incorporates an additional parameter to represent the exchange flux^26^. This enhanced version demonstrated superior performance in capturing nonlinear dynamics of catchments, particularly in predicting low-flow discharges^23,24^. The FLEX model leverages detailed hydrological processes, including infiltration, surface runoff, and groundwater flow, through the utilization of four distinct reservoirs and ten calibrated parameters, providing a more mechanistic representation of rainfall-runoff interactions^21,22^.

The lumped GR4H and GR5H models were implemented using the freely available R package, airGR^34^. The FLEX model was executed using Python, following the model description^22^. This simple conceptual approach requires only hourly precipitation, PET, and runoff series. The parameters of all three lumped models were calibrated based on maximizing the Nash-Sutcliffe efficiency criterion. To minimize the influence of initial state conditions, a preliminary warm-up period of one year was used at the beginning of the calibration dataset.

### Long short-term memory

LSTM architecture is a sophisticated model adept in handling sequence patterns, demonstrating superior performance compared to most of the RNN models across various applications^35,36^. The power of the LSTM model arises from its unique structure, which incorporates three specialized gates: the input gate, the forget gate, and the output gate^37^. These gates regulate the flow of information through the network, enabling the model to selectively retain or discard information over extended sequences. The typical structure of the LSTM model is defined according to the following equations:

1 $$\:{f}_{t}=\:\sigma\:\:({W}_{fx}{x}_{t}+\:{W}_{fh}{h}_{t-1}+\:{b}_{f})$$

2 $$\:{i}_{t}=\:\sigma\:\:({W}_{ix}{x}_{t}+\:{W}_{ih}{h}_{t-1}+\:{b}_{i})$$

3 $$\:{o}_{t}=\:\sigma\:\:({W}_{ox}{x}_{t}+\:{W}_{oh}{h}_{t-1}+\:{b}_{o})$$

4 $$\:{g}_{t}=\:\varphi\:\:({W}_{gx}{x}_{t}+\:{W}_{gh}{h}_{t-1}+\:{b}_{g})$$

5 $$\:{C}_{t}=\:{g}_{t}\:\odot \:{i}_{t}\:+\:{C}_{t-1}\:\odot \:{f}_{t}$$

6 $$\:{h}_{t}=\:\varphi\:\:\left({\:C}_{t}\:\right)\:\odot \:{o}_{t}\:$$

where $\:\sigma\:\:and\:\varphi\:$ are the logistic sigmoid and tanh activation functions; $\:W$ represents the weight matrix for each gate; $\:b$ is the bias term; $\:\odot$ is the elementwise multiplication. $\:{x}_{t}$ is the time-series input at the *t*th step. $\:{C}_{t}$ represents the cell state core, which contains three gates $\:{f}_{t},\:{i}_{t}\:and\:{o}_{t}$ for forget, input and output gates, respectively. $\:{g}_{t}$ is the updated condition of the cell state; $\:{h}_{t}$ is the intermediate information flowing between the cells^35^.

#### GR4H-LSTM model

This study presents a novel technique that integrates the strengths of conceptual models with the advanced capabilities of LSTM networks to enhance runoff predictions. Specifically, we aim to improve the runoff predictions by using the outputs of the GR4H model as inputs to the LSTM model. The selection of the GR4H model was based not only on its widespread application in hydrological studies but also on its parsimonious structure, which facilitates robust performance even with limited calibration datasets. The proposed GR4H-LSTM model incorporates the hourly outputs of the GR4H model along with three key inputs: hourly precipitation, hourly PET, and accumulated precipitation over the preceding 24 h. This approach is designed to optimize efficiency while capturing both short-term and long-term dependencies between the inputs and outputs, using an IDL of 50 h. The GR4H-LSTM model improves the network’s performance by extracting precise information from an informative and limited set of inputs, thereby optimizing the network’s performance.

GR4H-LSTM model is developed with a focus on efficiency, encompassing only three layers. The model consists of an initial LSTM layer with 32 units, followed by a dropout layer with a rate of 0.2 to reduce overfitting. Subsequent layers include a dense layer with 16 units featuring linear activation, intended to facilitate a seamless information transition to the output layer, which comprises a single neuron for continuous target prediction. The model is compiled with the Adam optimizer and Mean Squared Error (MSE) as the loss function, designed to minimize prediction errors in a regression framework^38^.

#### Multi-scale LSTM model

This study proposes another version of the LSTM that operates independently of conceptual models, functioning as a stand-alone model. This model is designed to enhance efficiency, by leveraging a limited set of informative inputs. The novelty of this approach lies in the integration between coarse and fine temporal Multi-Scale inputs into an LSTM architecture, termed as MS-LSTM, to capture both long and short dependencies between the inputs and outputs. To enhance efficiency, the MS-LSTM model is designed to avoid using finer temporal resolution inputs for capturing long-term hydrological processes, instead employing coarse inputs. Conversely, finer resolution inputs are essential for accurately capturing short-term hydrological dependencies. This approach necessitates the implementation of a gradual multi-scale input strategy, encompassing finer resolution data to capture short-term hydrologic behavior closer to the simulated time step, while simultaneously incorporating coarser resolution inputs to account for the long-term impacts inherent in hydrological processes.

**Fig. 2:**
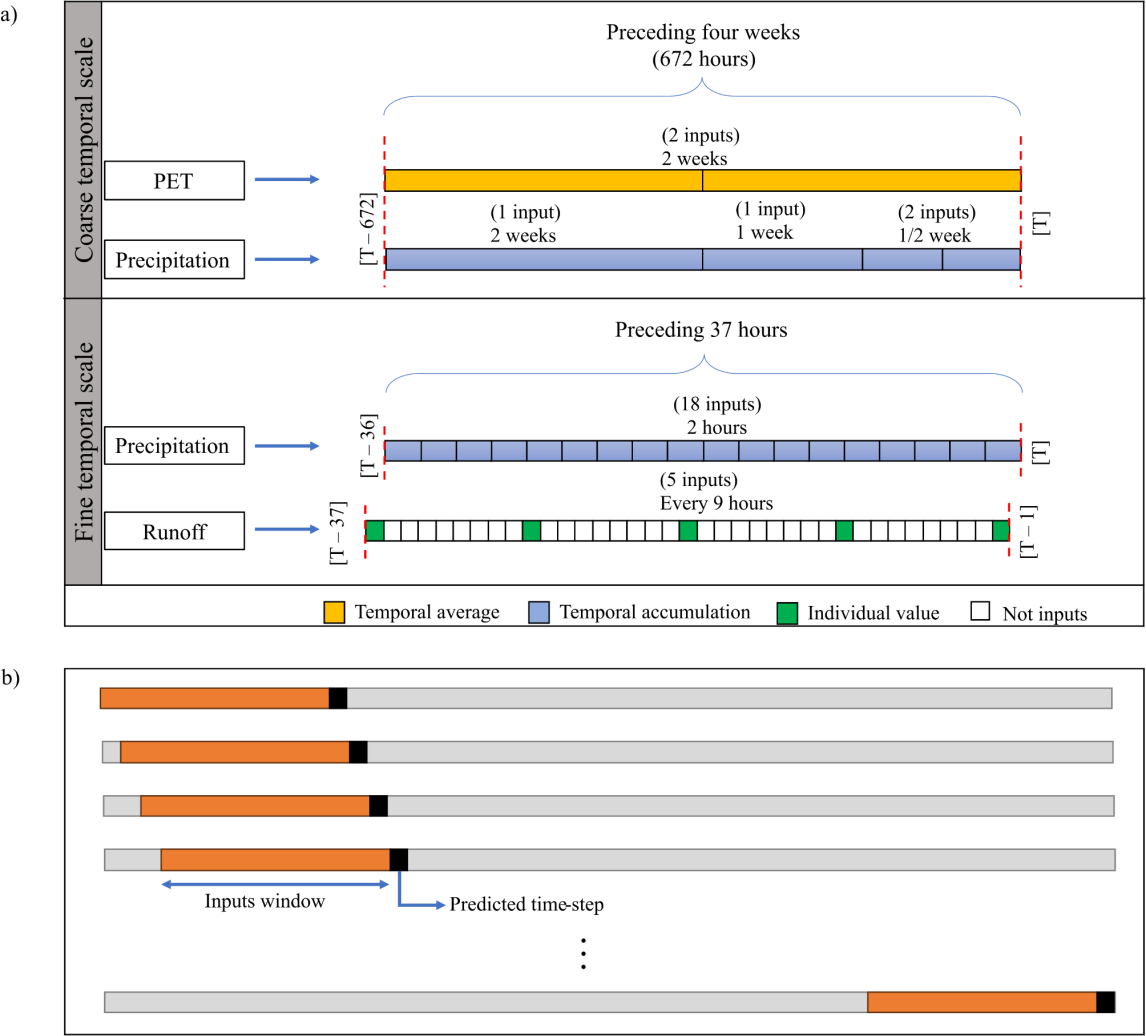
MS-LSTM model: (**a**) model inputs, and (**b**) the testing technique: one-step predicted runoff is used as an input for the following time steps.

MS-LSTM requires three distinct features: precipitation, PET, and preceding runoff responses, as represented in Fig. 2a. To represent PET, two average values are used for the first and second two weeks of the month preceding the simulated time step. This aims to avoid the complexity of employing a large number of high-resolution PET inputs, which do not significantly enhance accuracy. In contrast, the precise representation of precipitation characteristics necessitates both coarse and high-resolution inputs. The model integrates four coarse-scale inputs to represent precipitation over the preceding month, including accumulated precipitation values over 2 weeks, 1 week, and two half weeks. Additionally, it incorporates 18 high-resolution inputs, each corresponding to the accumulated precipitation over a 2-h interval. Furthermore, the hydrologic nature of discharge responses in streams follows a sequential pattern, where incorporating lagged information proves effective in predicting the following runoff responses. Therefore, the model utilizes five preceding hourly runoff values, with one runoff value every 9-hr. Ultimately, the total number of inputs is 29 (2 for PET, 22 for precipitation, and 5 for runoff), representing a significant reduction in the inputs required compared to other existing machine learning models, which may require thousands of inputs.

The MS-LSTM model is developed with a focus on efficiency, comprising only three layers. The model starts with an LSTM layer, followed by two fully connected layers. The LSTM layer has 16 hidden units to capture the temporal dependencies, utilizing the Gaussian Error Linear Unit (GELU) activation function for enhanced gradient propagation^39^. The first dense layer, consisting of 6 neurons, also utilizes the GELU activation function to maintain non-linearity throughout the network. The final dense layer, featuring a single neuron with an implicit linear activation, is designed to output a singular runoff prediction value. This configuration demonstrates a balance between model complexity and performance, facilitating effective runoff predictions while maintaining reasonable computational demands during both the training and prediction stages. The model is compiled with the Adam optimizer, employing the MSE as a loss function^38^.

At the training stage, the MS-LSTM utilizes preceding reference runoff values as inputs to predict the next runoff value, as shown in Fig. 2a. While incorporating lagged information during model training is common and acceptable for sequential models, it is crucial to verify the model’s capability to function independently in real-world scenarios without relying on such preceding data. To address this concern and prevent any potential data leakage during the testing stage, a one-step prediction technique is investigated. This technique involves executing the model iteratively, as illustrated in Fig. 2b, wherein the model’s predictions serve as inputs for subsequent time steps. Specifically, after the initial prediction using observed data, each subsequent forecast utilizes the previous time step’s prediction as an input, rather than observed values. This iterative process continues throughout the testing period, with the model generating and using its own predictions as inputs. Effectively, this process enables the model to generate its own future inputs, simulating autonomous operation in practical applications and assessing the model’s ability to maintain accuracy over extended periods without continuous access to observed data.

### Performance evaluation

In this study, several performance criteria are used to compare between the simulated and reference hourly runoff series. The Nash-Sutcliffe efficiency (NSE) index^40^, Kling-Gupta efficiency (KGE) criterion^41^, the coefficient of determination (R^2^)^42^, and the Root Mean Squared Error (RMSE)^43^ have been widely used in the water resources sector to compare between simulated and observed series^25,44,45^, according to the following equations:

7 $$\:NSE=1-\frac{{\sum\:}_{i=1}^{n}{({R}_{i}-{S}_{i})}^{2}}{{\sum\:}_{i=1}^{n}{({R}_{i}-\stackrel{-}{R})}^{2}}$$

8 $$\:KGE=1-\sqrt{{(r-1)}^{2}+{(\beta\:-1)}^{2}+{(\alpha\:-1)}^{2}}$$

9 $$\:{R}^{2}=1-\frac{{\sum\:}_{i=1}^{n}{\left({R}_{i}-{S}_{i}\right)}^{2}}{{\sum\:}_{i=1}^{n}{\left({R}_{i}-\stackrel{-}{R}\right)}^{2}}$$

10 $$\:RMSE=\sqrt{\frac{1}{n}{\sum\:}_{i=1}^{n}{({R}_{i}-{S}_{i})}^{2}}$$

where $\:{R}_{i}\:and\:{S}_{i}$ are the reference and the simulated values respectively; *r* is the Pearson correlation coefficient between simulated and reference series; *β* is the ratio between the mean of the simulated and reference series; *α* is the ratio between the standard deviation of the simulated and reference series; $\:\stackrel{-}{R}$ is the average value of the reference series; and *n* is the number of data points in the reference series.

Flow Duration Curves (FDCs) are essential tools for providing a comprehensive representation of streamflow variability and facilitating informed decision-making across various hydrological applications such as water-use planning, hydropower management, flood control, dam operations, water-quality management, and geomorphological studies. Therefore, it is important to evaluate the models’ performance in capturing the reference FDC. The Average Absolute Deviation (AAD) is investigated to compare between the predicted and reference FDCs^29^, as defined in Eq. (11). AAD values range from 0 to ∞, with lower values closer to 0 indicating a better match. To estimate a representative AAD value between FDCs, for both high runoff and baseflow responses, it is recommended to investigate a wide range of exceedance probabilities: 0.001%, 0.01%, 0.1%, 0.5%, 1%, 4%, 10%, 25%, and 50%.

11 $$\:AAD=\frac{1}{n}{\sum\:}_{i=1}^{n}\frac{\left|{Q}_{{p}_{i}\text{\%}}^{r}-{Q}_{{p}_{i}\text{\%}}^{S}\right|}{{Q}_{{p}_{i}\text{\%}}^{r}}$$

where $\:{Q}_{{p}_{i}\%}^{r}\:and\:{Q}_{{p}_{i}\%}^{S}$ are the runoff values at the exceedance percentage of $\:{p}_{i}\%$ for reference and simulated series, respectively. The investigated number of various exceedance percentages is denoted by $\:n$.

## Results and discussion

This study primarily assesses the impact of the training dataset duration on the performance of the models, while comparing conceptual and advanced DL approaches in their capacity to capture the temporal dynamics of hydrological responses. To enhance the fidelity of this study, a century-long dataset was used, offering high-resolution precipitation, PET, and runoff responses at an hourly timescale. The first 50-year period of the dataset was allocated for calibration and training purposes, utilizing various sub-periods datasets. The testing dataset, comprising the latter half of the century-long dataset, remained constant across all simulation iterations to ensure consistent evaluation.

### Impact of calibration dataset duration on model performance

**Fig. 3:**
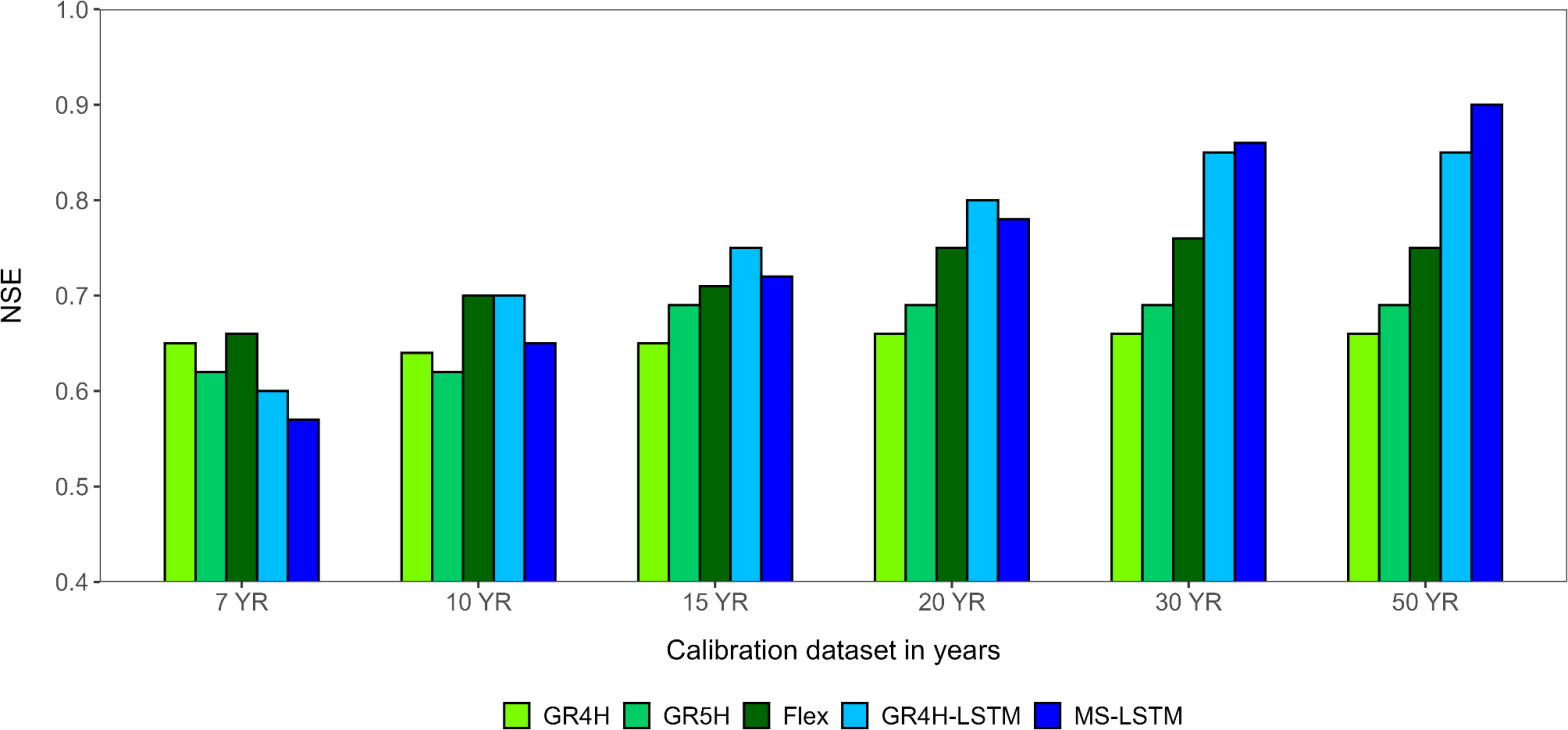
Comparison of validated NSE values with increasing the training period length for both conceptual and LSTM models.

The impact of varying training data lengths on the accuracy of runoff response predictions by conceptual and DL models was investigated. Figure 3 presents the testing NSE values derived from five models as the training dataset length increases from 7 to 50 years. The improvement in validation NSE values between the top-performing models of both DL and conceptual approaches is 6%, 13%, and 20% for training datasets of 15, 30, and 50 years, respectively. This comparison resulted in the following observations:

- Conceptual models demonstrate superior performance relative to the developed LSTM models when trained on a 7-year dataset. However, both approaches exhibit similar performance at the 10-year dataset. At the 15-year duration and longer, both LSTM models consistently surpass the performance of all three conceptual models.
- The performance of both LSTM models demonstrates a continuous improvement trend as the length of the training dataset increases. Conversely, there is no significant enhancement observed in the performance of conceptual models beyond a training dataset length of 15 years.

The more parsimonious conceptual models indicate robust performance at shorter training dataset lengths. However, as the complexity of the models increases, their predictions improve with longer training periods.

The GR4H-LSTM model outperforms MS-LSTM at short to medium training dataset lengths, suggesting that the simplified conceptual background of GR4H is more effective with limited data-series lengths. However, beyond a training dataset length of 30 years, MS-LSTM begins to exhibit superior performance. This indicates the effectiveness of employing gradual multi-scale inputs, including both coarse and fine temporal inputs, to overcome the lack of physics-based information.

**Fig. 4:**
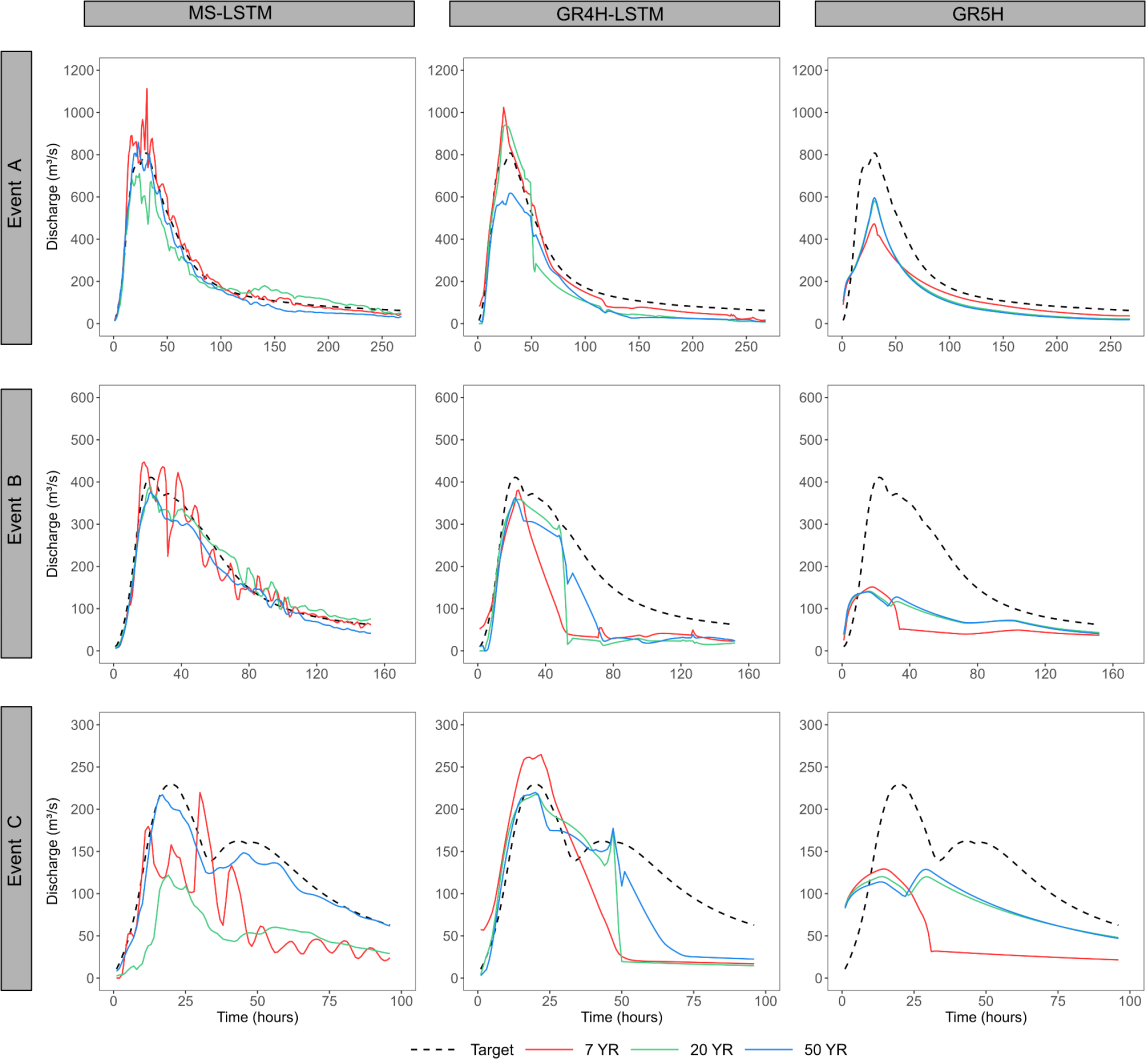
Reference and predicted hydrographs for three example events with increasing the calibration period length for three models: MS-LSTM, GR4H-LSTM, and GR5H.

The performance evaluation of the models was further extended by comparing the predicted with reference time series. To achieve this, three distinct events representing floods of varying magnitudes were selected to illustrate how the performance of the models evolves with increasing training dataset lengths, as illustrated in Fig. 4. The key findings are outlined as follows:

- Both LSTM models significantly outperform the GR5H model in simulating peak flow responses and the recession limb of hydrological events.
- The MS-LSTM model exhibits significant instability in runoff predictions when trained on a short 7-year dataset. However, this instability diminishes, and the model becomes more robust with longer training datasets. This is evident in the increasing alignment between the predictions and the reference curve, particularly for Events A and C.
- The GR4H-LSTM model consistently produces robust runoff predictions and accurately captures peak flows, especially with longer training datasets. However, it faces limitations in precisely modeling the recession limb of flood events, especially for Events B and C.
- The GR5H model consistently underestimates peak flows across all three events. Longer training datasets demonstrate no noticeable improvement in runoff predictions, indicating the model’s limited capacity to effectively learn from the provided data.

### Comparative analysis: conceptual vs. LSTM models over 50-Year training

**Fig. 5:**
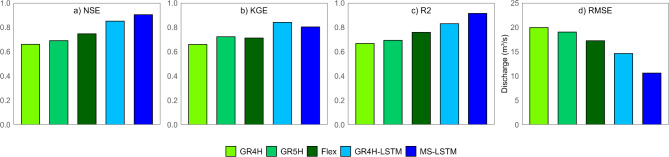
Validation portion evaluation for using both conceptual and LSTM models according to various performance criteria: (**a**) NSE, (**b**) KGE, (**c**) R^2^, and (**d**) RMSE.

The performance of the models was evaluated using a 50-year training dataset, employing various performance criteria to assess their ability to predict the reference hydrographs. The DL models demonstrate superior performance over the conceptual models across all examined criteria, as illustrated in Fig. 5. The MS-LSTM model achieves the highest performance with NSE and R^2^ of 0.90 and 0.91, respectively. The GR4H-LSTM model, however, achieved the highest KGE score of 0.84. Conversely, the simplicity of the conceptual models constrained their ability to leverage the advantages of long training datasets, thereby limiting their performance in accurately predicting hydrological responses. The GR4H model, with the simplest structure, exhibits the lowest accuracy, with an NSE value of 0.66 and the highest RMSE approaching 20 m^3^/s.

**Fig. 6:**
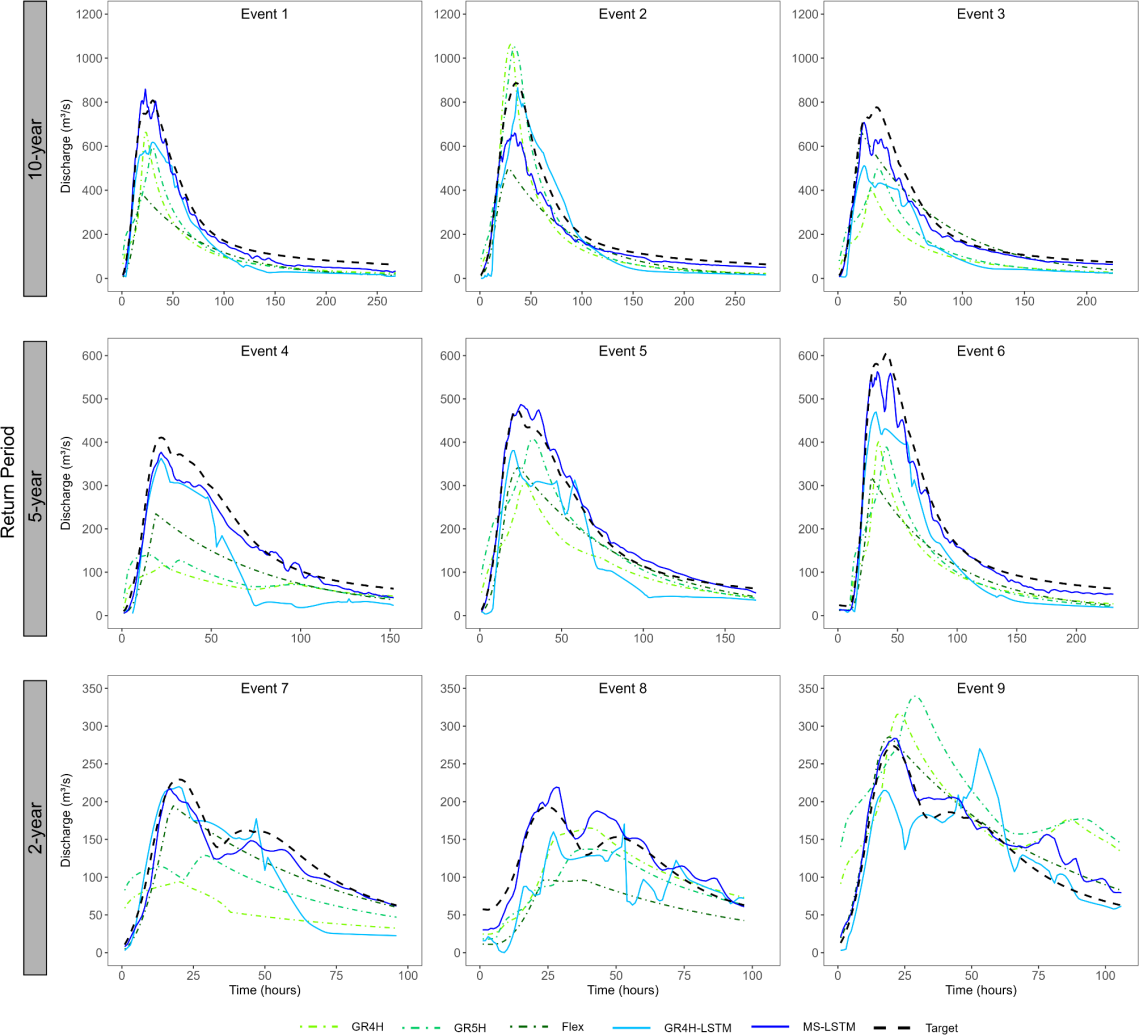
Reference and predicted hydrographs for various example events using both conceptual and LSTM models (validation portion).

Evaluating model performance across a spectrum of hydrological events is crucial for assessing the robustness and versatility of predictive models. Figure 6 presents time-series comparisons between reference and predicted values for nine distinct events. These events span a range of return periods, from relatively frequent occurrences (2-year return period) to more rare events exceeding a 10-year return period. The following key observations can be drawn from this comparison:

- While no single model demonstrates robust performance across all investigated events, both LSTM models exhibit consistent performance with less deviation compared to the conceptual models.
- Conceptual models generally tend to underestimate the peaks of the investigated events, with exceptions observed in Events 2 and 9, where they notably overestimate the peak responses.
- The MS-LSTM model demonstrates strong capabilities in capturing the peaks, except for Event 2, where predictions are notably underestimated.
- The GR4H-LSTM model performs well in predicting the runoff responses, although it tends to underestimate peaks, especially at Events 3, 5, and 6.

**Fig. 7:**
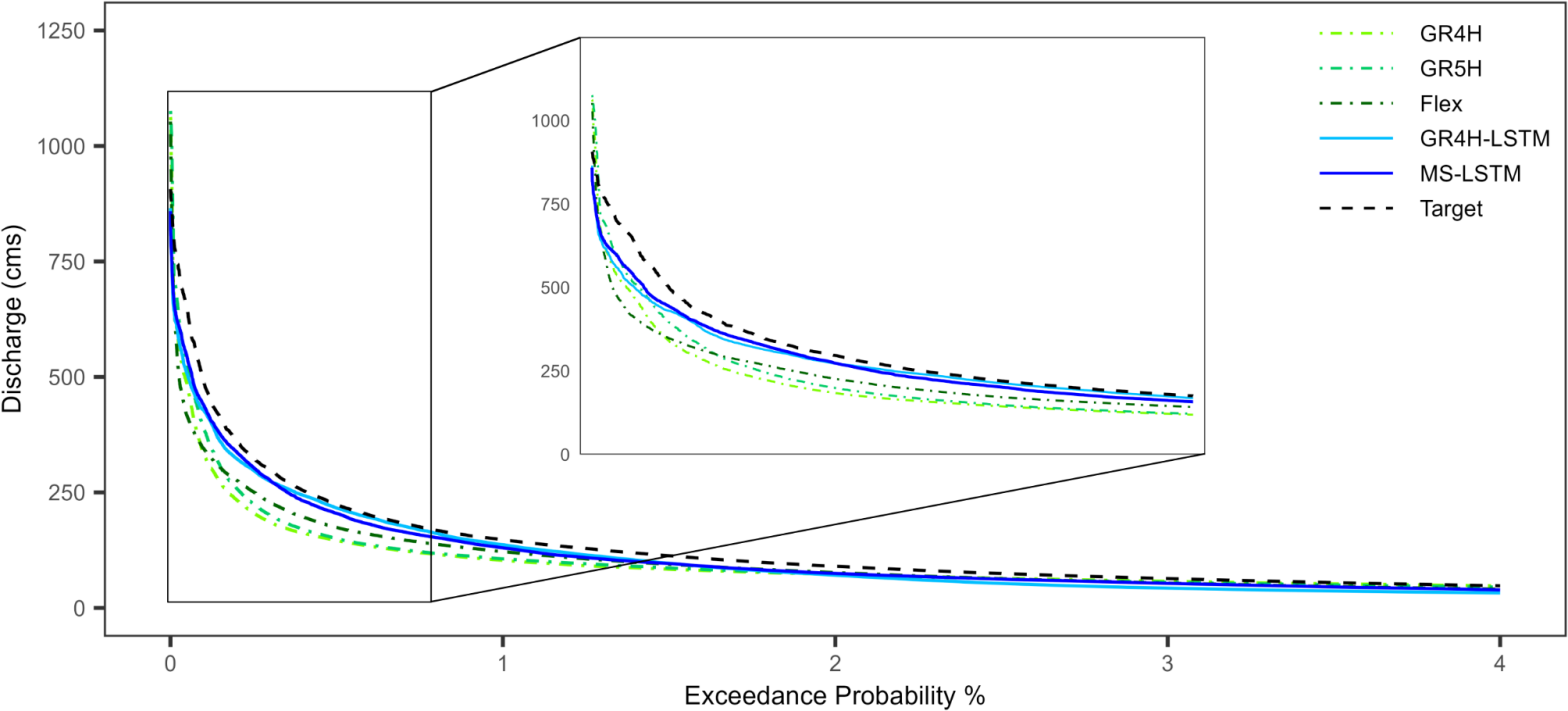
Comparison of FDCs for the reference series against the predicted series using both conceptual and LSTM models (validation portion).

The evaluation of model performance in reproducing FDCs is essential for a comprehensive assessment of their capabilities, as FDCs are widely utilized to characterize overall runoff patterns in various water resource planning and management applications. Figure 7 compares the FDCs predicted by both DL and conceptual models against the reference series, with a specific focus on the highest 4% exceedance probability. This comparison yields several significant insights into model performance and applicability:

- The conceptual models exhibit limited accuracy in capturing the FDC, particularly at the highest 1% exceedance probability, compared to the LSTM models.
- Both LSTM models demonstrate enhanced accuracy in representing the maximum peak of the reference FDC, outperforming the conceptual models, which exhibit a significant tendency to overestimate this critical value.
- The GR4H-LSTM model demonstrates the least deviation with an AAD value of 13.5%, whereas the GR4H model has the highest value of approximately 24%.

## Conclusions

This study evaluated three conceptual and two novel LSTM models for predicting hourly runoff responses based on several performance criteria. To optimize computational efficiency in hydrological simulations, both LSTM models were developed with simple structures and minimal input requirements. The GR4H-LSTM model represents a novel technique to incorporate physics-based knowledge into the DL framework by employing the outputs of the GR4H model as inputs into the LSTM model, in addition to traditional inputs: precipitation and PET. This proposed architecture extracts valuable insights from the conceptual model, significantly enhancing prediction accuracy within an efficient framework by requiring substantially fewer IDL inputs. However, it shows limitations in precisely capturing the recession limb of flood events.

The second model, MS-LSTM, was designed as a stand-alone model relying on the precipitation, PET, and preceding runoff responses. The model’s applicability was rigorously tested through an iterative process, where runoff predictions from each time step were used as inputs for subsequent forecasts, demonstrating the model’s capacity to maintain accuracy over extended periods for real-world scenarios without relying on continuous observed data. This model employs a gradual multi-scale input strategy, encompassing finer and coarse resolution inputs, aiming to achieve two key objectives: (a) minimizing the number of required inputs, and (b) capturing both long-term and short-term dependencies within the data. By utilizing a limited yet informative set of inputs, the model facilitates less complex LSTM architectures. This simplicity enables the LSTM model to focus more effectively on the most relevant temporal dependencies, thereby enhancing the robustness and accuracy of its predictions. However, this model necessitates a relatively long training dataset to ensure reliable runoff predictions.

A synthetic meteorological dataset, generated from an advanced physics-based model and spanning 100 years, was employed in this study. This dataset not only facilitates the assessment of the impact of increasing training dataset periods on model performance but also provides a cleaner representation of rainfall-runoff processes, free from the noise and measurement errors inherent in real-world observations. Some key findings and recommendations from this study include the following:

Conceptual models exhibit superior performance compared to advanced DL models when training datasets are limited to 10 years or less. However, these conceptual models demonstrate limited capacity for improvement with longer calibration datasets beyond 15 years.

- Conceptual models exhibit superior performance compared to advanced DL models when training datasets are limited to 10 years or less. However, these conceptual models demonstrate limited capacity for improvement with longer calibration datasets beyond 15 years.
- Both LSTM models show a consistent improving trend in performance with increasing training dataset lengths, indicating superior learning capacity.
- The integration of LSTM capabilities with the basic GR4H model demonstrates significant potential. This hybrid approach achieves notably superior performance with a relatively short 15-year training dataset, with continued improvements observed as dataset length increases.
- The GR4H-LSTM outperforms the stand-alone MS-LSTM version for short to medium-length training datasets, underscoring the promising potential of integrating conceptual models with DL algorithms. The MS-LSTM compensates for the absence of the physical framework provided by the GR4H model by employing a longer IDL. This adaptation enables the MS-LSTM to capture the hydrological processes, although it requires longer training datasets to surpass the performance of the GR4H-LSTM model.
- Both LSTM models surpass conceptual models in predicting the reference FDC, particularly in the highest 1% exceedance probability. This enhanced accuracy in FDC prediction is crucial for effective water resource planning and management practices.

Utilizing data from physics-based models, rather than direct observations, is often constrained by the limited duration of observational data series. Further, while two distinct DL models based on LSTM were presented, future research is needed to evaluate the applicability and performance of other models, including Transformer and Gated Recurrent Units. Despite the potential of the GR4H-LSTM model, which requires outputs from a conceptual model, there is a need to develop a physics-informed ML model that can operate efficiently as a stand-alone tool without the need to obtain data from other conceptual models/tools. This integration of physics principles with ML techniques has the potential to address challenges associated with observed data, enabling more generalizable predictions.

## Data Availability

The raw data supporting the findings of this study were obtained from (Fathi et al., 2024), and are available from the corresponding author upon reasonable request.
